# Strangulated Falciform Hernia

**DOI:** 10.7759/cureus.15898

**Published:** 2021-06-24

**Authors:** Nagarajan Raj Kumar, Muhamed Tajudeen

**Affiliations:** 1 Surgery, Jawaharlal Institute of Postgraduate Medical Education and Research (JIPMER), Puducherry, IND

**Keywords:** falciform ligament hernia, strangulated hernia, murphy's sign, surgical acute abdomen, pyoperitoneum

## Abstract

Internal hernias are rare, and a delayed diagnosis can lead to dangerous complications. A 75-year-old male with no previous surgical history presented with right upper abdominal pain and vomiting. On examination, he had guarding in the right hypochondrium with a positive Murphy’s sign. However, ultrasonography of the gall bladder was normal with dilated bowel loops. Contrast-enhanced CT (CECT) revealed a falciform hernia with evidence of obstruction. Segmental resection of the gangrenous ileum was done with a double-barrel stoma. Later on, stoma reversal was also done with no complications.

## Introduction

An internal abdominal hernia is an abnormal protrusion of the abdominal organs through a hole in the mesentery or peritoneum, which can be congenital or iatrogenic without any hernia sac. Falciform ligament hernias are a rare type of internal hernia that occurs through a defect in the ligament. More often, falciform hernias tend to obstruct, and the preoperative diagnosis of falciform hernia is difficult. Resection of the bowel segment may be required if the herniated bowel loop is strangulated. Early intervention can prevent mortality in such cases. We report a case of strangulated falciform hernia, successfully managed with an outline of the literature.

## Case presentation

We present a case of a 75-year-old gentleman, a known case of diabetes, hypertension, and chronic kidney disease who presented with sudden onset colicky abdominal pain for two days in the right hypochondrium, associated with five episodes of bilious vomiting and abdominal distention. He had obstipation for two days. He had no prior abdominal surgeries and never had such similar episodes in the past. General examination was normal, and he was hemodynamically stable. Abdominal examination revealed a distended abdomen with tenderness and guarding in the right hypochondrium and had a positive Murphy’s sign. His bowel sounds were sluggish. The digital rectal examination had a stain of feces.

**Figure 1:**
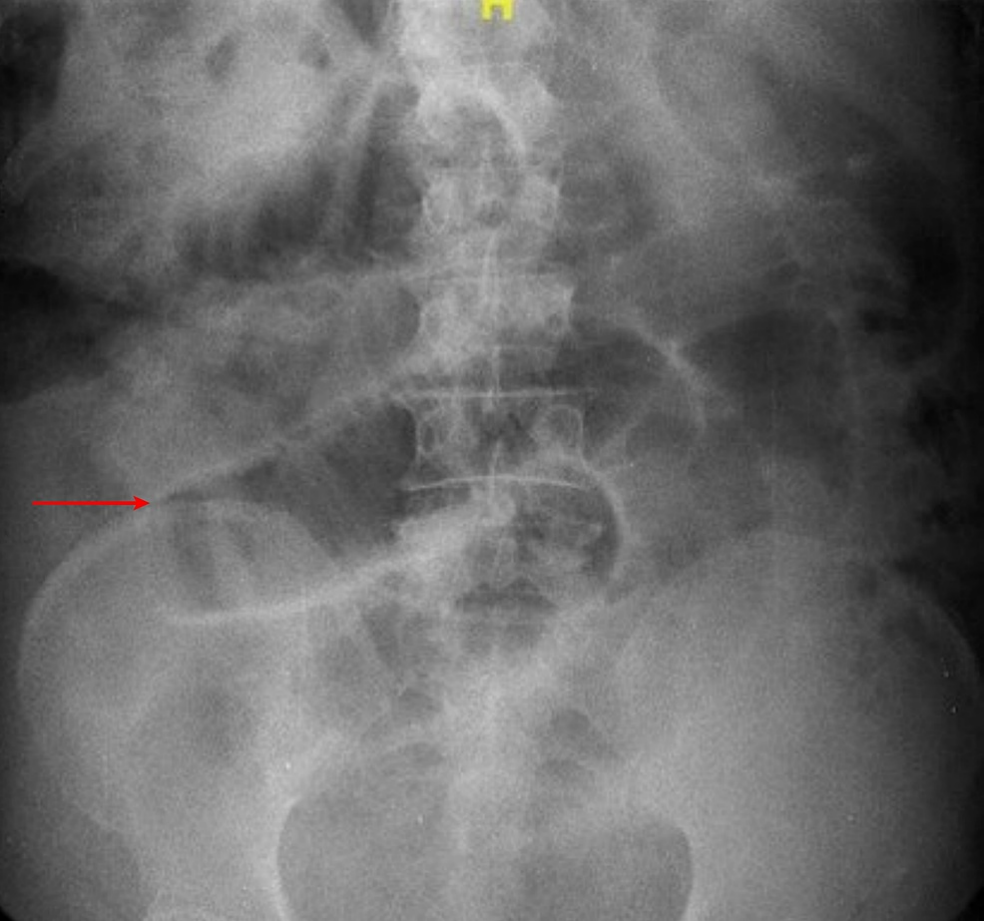
X-ray of the abdomen showing dilated jejunal loops (red arrow).

Blood investigations were normal. Suspicion of acute cholecystitis was in mind. However, ultrasonography revealed a normal gallbladder with dilated and sluggishly peristaltic small bowel loops with mild ascites. X-ray abdomen showed dilated jejunal loops (Figure 1).

**Figure 2:**
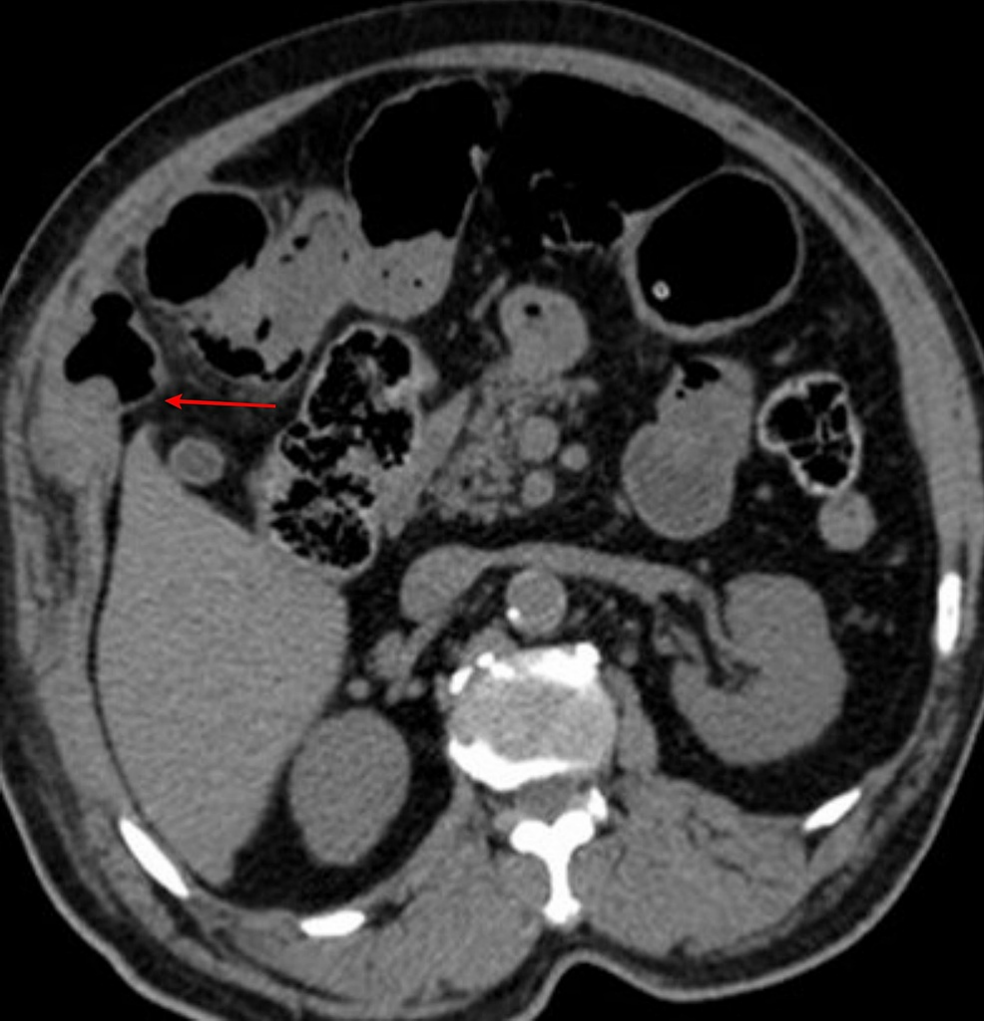
CECT of the abdomen showing herniation of ileal loops anterior to the liver (red arrow). CECT, contrast-enhanced CT

Contrast-enhanced CT (CECT) of the abdomen revealed herniated ileal loop through the parietal wall anterior to the liver with dilatation of proximal small bowel loops (Figure 2).

Ryle’s tube drained around 700 mL of bile. The patient was taken up for emergency laparotomy. Intraoperatively, there was pyoperitoneum with an ileal loop herniated from left to right through a defect in the falciform ligament of size 2 cm × 3 cm and was gangrenous for a length of around 10 cm for which segmental resection of the bowel with double barrel ileostomy was done in view of edematous bowel. No bands were noted. The defect in the falciform ligament was laid open. The postoperative period was uneventful, and the patient was discharged. After three months, ileostomy reversal was done and was uneventful.

## Discussion

Based on the type of hernial orifice, internal hernias can be classified into three categories. Firstly, through the normal foramen as epiploic foramen hernia. Secondly, through the peritoneal fossa like para-cecal hernia and paraduodenal hernia. And lastly through an abnormal opening in the mesentery or peritoneal ligaments like falciform ligament hernia and broad ligament hernia [1]. Of all, paraduodenal hernia is the most commonly encountered internal hernia [2]. Falciform ligament hernias account for 0.2% of all internal hernias. The presentation can be acute with signs of bowel obstruction or with nonspecific complaints. One symptom attributed to falciform hernia is that the pain relieves on the knee-chest position from the supine position [3].

Contrast-enhanced CT diagnoses internal hernias preoperatively in patients with obstruction. It can provide a definitive diagnosis of the type and content of the hernia along with the vascularity of the bowel. Suspicion of falciform hernia is considered if a closed-loop of the intestine is seen in front or slightly caudal to the liver. A dilated intestinal loop between the liver and the abdominal wall should provoke suspicion of falciform hernia [4]. In a review of 37 cases of falciform ligament herniation by Egle et al., only five were diagnosed preoperatively, emphasizing the diagnostic difficulty. It is most often an intraoperative diagnosis at times of surgical exploration for obstruction. Most falciform ligament hernias present with obstruction requiring resection and anastomosis in nearly 50%. The falciform defects can either be congenital or traumatic. Of late, most are due to trocar injury during the laparoscopic approach [5].

In an era of domination by minimal access surgery, firstly, internal herniation can be prevented by slight tweaks in the technique by placing the subxiphoid trocar just to the right of the midline in laparoscopic cholecystectomy, avoiding falciform injury. Secondly, even if the aperture is created in the falciform, division of the falciform ligament, including the round ligament, can prevent herniation. Finally, removal of the subxiphoid port under vision prior to the de-sufflation of the pneumoperitoneum can prevent the postoperative chances of herniation.

## Conclusions

A falciform ligament herniation is a significantly morbid condition considering the chances of obstruction. Always morbidity is better prevented than treated. In this era of laparoscopic surgery, a few slight modifications of techniques can prevent such significant morbidity that can threaten a patient’s life. Knowledge of the falciform hernias and a high clinical suspicion is generally required so as to intervene early in the patients.
